# Bacteriophage Intervention in Pseudomonas aeruginosa-Induced Corneal Ulceration: A Multifaceted Study Assessing Therapeutic Efficacy in Surrogate Bacterial and Animal Models

**DOI:** 10.7759/cureus.108721

**Published:** 2026-05-12

**Authors:** Aalok Kumar, Lavanya Anuranjani, Mayank Gangwar, Arup Dey, Gopal Nath

**Affiliations:** 1 Department of Ophthalmology, Institute of Medical Sciences, Banaras Hindu University, Varanasi, IND; 2 Department of Obstetrics and Gynaecology, Institute of Medical Sciences, Banaras Hindu University, Varanasi, IND; 3 Department of Development Research, Clinical Studies and Trial Unit, Indian Council of Medical Research (ICMR), New Delhi, IND; 4 Department of Pharmaceutical Technology, School of Health and Medical Science, Guwahati, IND; 5 Department of Microbiology, Institute of Medical Sciences, Banaras Hindu University, Varanasi, IND

**Keywords:** bacteriophage therapy, corneal ulceration, pseudomonas aeruginosa (p. aeruginosa), rat model study, therapeutic intervention efficacy

## Abstract

Introduction: The aim of our study was to learn about the effect of bacteriophages derived from the water of the river Ganga on *Pseudomonas*-induced corneal ulcer in mice. *Pseudomonas aeruginosa *poses a significant threat in cases of bacterial keratitis, a condition that severely hampers vision. With variable incidence rates worldwide and increasing antibiotic resistance to *Pseudomonas*, it is necessary to explore the potential of bacteriophages isolated from Ganga water for treating *Pseudomonas aeruginosa*-induced keratitis.

Material and methods: We used a mouse model for this study. The most potent phages targeting *Pseudomonas aeruginosa *that demonstrated a broad spectrum of activity against clinical strains were phenotypically and genotypically characterised. Establishing the animal model involved inducing severe keratitis in mice by inoculating them with *Pseudomonas aeruginosa*. After this, we administered multiple doses of bacteriophages as eye drops, 24 hours after infection.

Results: The results yielded significantly improved disease outcomes. The treatment not only preserved the structural integrity and transparency of the infected cornea but also suppressed neutrophil infiltration and bolstered bacterial clearance, as evidenced by histopathological analysis.

Conclusion: These findings suggest that bacteriophage therapy can be an effective alternative therapeutic approach for treating antibiotic-resistant infectious keratitis. Also, we can use bacteriophages in combination with antibiotics already in use.

## Introduction

The cornea, which has intricate and well-organised optical properties, is the primary refractive medium of the visual system [1]. The tear film, a complex mixture of lipids, proteins, mucins, defensins, and electrolytes, keeps the ocular surface, including the cornea, smooth and moist [2]. Numerous species of potentially harmful bacteria, such as *Pseudomonas*, *Corynebacterium*, *Acinetobacter*, *Staphylococcus*, and *Streptococcus*, are present on the ocular surface [3]. Being transparent and avascular, the cornea plays an important role in refraction and final visual acuity. The appropriate spacing of the collagen fibres in the corneal stroma is the main factor in its transparency. To prevent tissue damage and scarring from infectious corneal ulcers, which can also threaten vision, timely diagnosis and adequate treatment are essential. *Pseudomonas aeruginosa* is one of the most common causes of keratitis, particularly among individuals who wear contact lenses [4]. *Pseudomonas aeruginosa* causes ulcers rapidly and is characterised by tissue damage and inflammatory cell infiltration, which may lead to corneal perforation. Mostly, antibiotics are commonly used to eradicate the infecting organisms. However, methicillin-resistant *Staphylococcus aureu*s (MRSA), multidrug-resistant *Pseudomonas aeruginosa*, and vancomycin-resistant *Enterococcus *(VRE) are examples of antibiotic-resistant bacterial infections that have become a significant clinical issue in recent years. Multidrug-resistant bacteria, including MRSA, frequently invade the ocular surface [5]. Therefore, it is necessary to discover a novel complementary or alternative medicine for the management of bacterial ulcers. Among these are phages, also known as viruses, which were discovered over 80 years ago and are found in all parts of the environment, including food, water, soil, and the gastrointestinal system [6]. Phages cause no harm to mammalian cells as they infect, lyse, and destroy bacteria. Phage therapy was therefore quickly considered a possible treatment for bacterial infections [7]. Phage treatment is therapeutically effective in in vivo studies [8].

## Materials and methods

We used a mouse model for our study, for a duration of two years (March 2023 to March 2025). A *Pseudomonas aeruginosa* strain was isolated and identified from various clinical samples of the Bacteriology section, Department of Microbiology, Institute of Medical Sciences (IMS), Banaras Hindu University (BHU), Varanasi, India. The bacteriophage was isolated against the *Pseudomonas* bacterial strain 11434. The identified *Pseudomonas aeruginosa* strains were processed for confirmation and differentiation. All chemicals and reagents were taken from the Virus Research and Diagnostic Laboratory at the Department of Microbiology, IMS, BHU, Varanasi. Mueller-Hinton agar (MHA) medium was used for bacterial culture, counting colony-forming units (CFU), and the isolation of bacteriophages. Soft agar was used for phage-plaque formation. 

**Figure 1:**
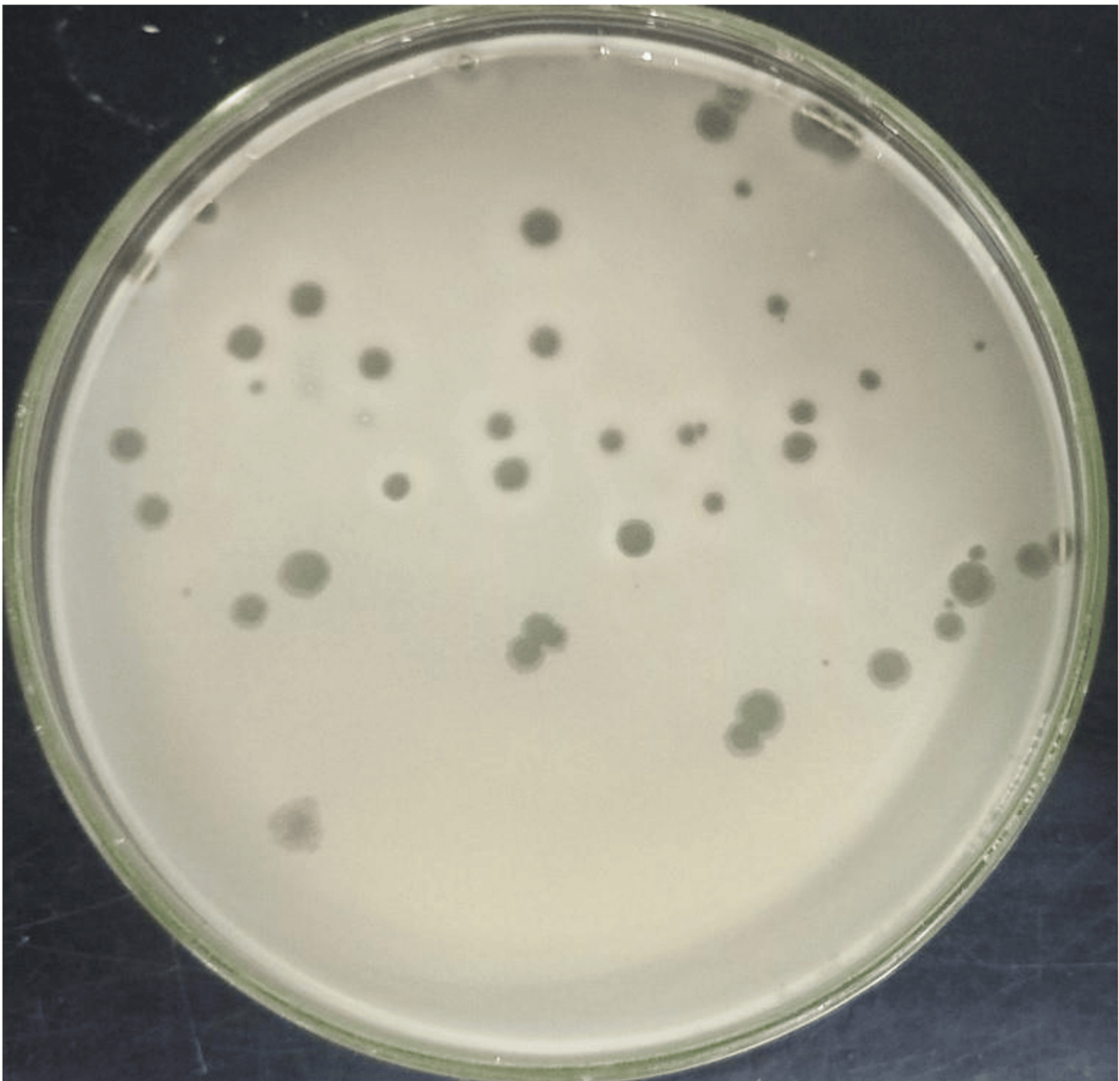
Activity of bacteriophages against the Pseudomonas aeruginosa strain on a Mueller-Hinton agar plate

Bacteriophages were isolated from water samples collected from different ghats along the river Ganga in Varanasi. The main site of our water collection was the Ganga water from near Dashashwamedh Ghat, Manikarnika Ghat, and Namo Ghat. Using the *Pseudomonas aeruginosa *strain 11434 as a host, bacteriophages were isolated, and host specificity was assessed using a 5 μL phage suspension dropped onto an MHA plate containing the *Pseudomonas aeruginosa* strain and incubated at 37°C overnight. After incubation, the infected area was characterised into three categories: clear plaques, turbid plaques, and faint plaques, as shown in Figure 1.

Once the confluent plaques were obtained, they were used to produce bacteriophages in bulk. A single plaque was picked to increase the number of bacteriophages by repeatedly dropping the phage onto the bacterial lawn until the number reached 1012 plaque-forming units (PFU)/ml in the Roux bottle. 

Bulk production followed the removal of contamination from bacteriophages using a syringe filter (0.45-micron syringe filter) to remove large particles and contamination. The tris magnesium sulphate gelatin (TMG) buffer was then mixed with the harvested phage to dilute the bacteriophages. Then the sterile MHA Roux bottle was swabbed with the bacterial suspension and incubated at 37ºC for four hours. After four hours of incubation, the diluted phage was removed and dropped onto the pre-prepared bacterial lawn. Then, it was incubated at 37°C for one complete night. The next day, if a clear plaque appeared on the plate, the bacteriophages were collected in a microcentrifuge tube by washing the bottle surface with TMG buffer. The collected bacteriophage was then mixed with 1% chloroform, followed by an inversion method for 15 minutes. After this, it was then centrifuged at 10,000 rpm (rounds per minute) for 10 minutes. The supernatant was then collected and stored at 4°C. Then, it was purified using a syringe filter with a 0.22 μm pore size.

Bacteriophage genomic DNA was extracted with the QIAamp DNA Mini Kit (Qiagen, Hilden, Germany). Polymerase chain reaction (PCR) amplification was performed with Enterobacterial Repetitive Intergenic Consensus-Forward primer (ERIC-F) (5′-ATGTAAGCTCCTGGGGATTCAC-3′) and (5′-AAGTAAGTGACTGGGGTGAGCG-3′) primers. Each fingerprint was confirmed twice. Each ERIC-PCR test was performed twice to confirm each fingerprint.

**Figure 2:**
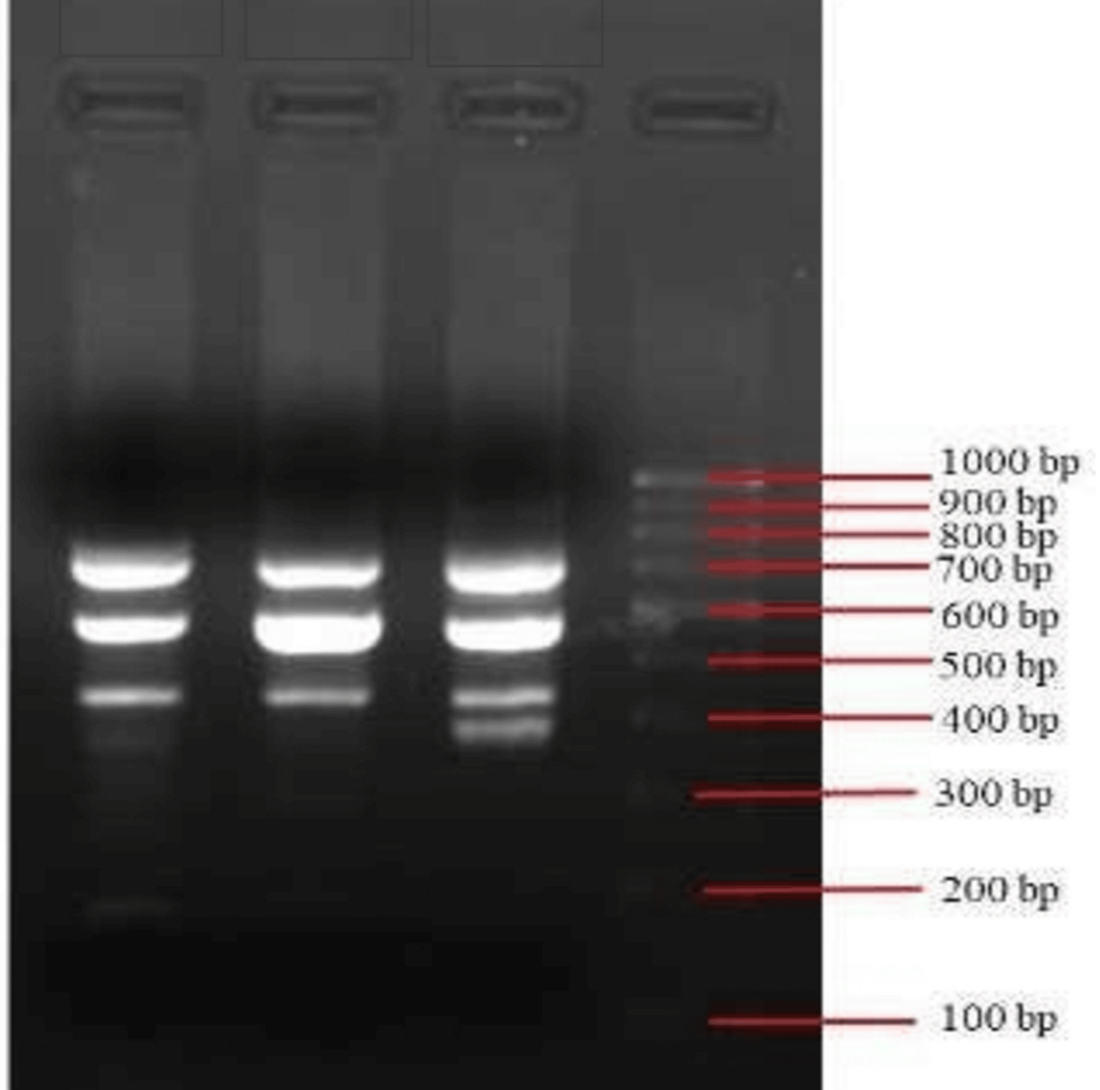
Gel electrophoresis bands of phage DNA

The DNA-binding patterns were entered into the Image Lab 2.0 Software (Bio-Rad Laboratories, Inc., Hercules, CA, USA) database to automatically obtain and analyse the image. Phages were classified as similar only when 80% or more of the bands were detected, as shown in Figure 2.

Twenty-four healthy Swiss albino mice of eight to 10 weeks were taken for this study from the Central Animal House (IMS, BHU, Varanasi). Approval for the project was obtained from the Central Animal Ethical Committee of the BHU before the experimental work (Notification no. Dean/2024/AIEC/6875, dated 20.01.2024).

Mice were anaesthetised 30 minutes before infection with an anaesthetic agent, ketamine (75-80 mg/kg body weight). Mouse eyes were infected using the protocol. Two to three scratches of 1 mm length were made on the cornea of one eye of every mouse using a sterile 25-gauge needle. A 5 μl of 2 optical density (OD) bacterial suspension containing *Pseudomonas aeruginosa* (11434) was applied to the corneal surface of each mouse to infect it. Twenty-four hours after infection, the 24 mice were randomly divided into four groups, each containing an equal number (six) of mice. The groups were named A, B, C, and D, respectively. Group A was marked as the infected control group and received phosphate-buffered saline (PBS) without phage as a blank treatment; Group B was marked as the positive control group, which received ciprofloxacin (0.3%) drops four times a day; and Groups C and D were marked as test groups with two concentrations of bacteriophage that were 4.6×10⁶ PFU/ml (phage 1) and 8.0×10⁹ PFU/ml (phage 2) PFU, respectively. 4.6×10⁶ PFU/ml and 8.0×10⁹ PFU/ml of phage in 5 μl or vehicle were applied to the corneal surface four times a day. The eyes were examined on day 1 and then every other day post infection to grade disease severity using an established scale, as described previously. The eyes were observed for corneal oedema.

After the scratch in the mouse's eye, a pus sample was collected daily with a sterile swab stick and stored in 1 ml of normal saline. The collected sample was then vortexed for uniform distribution. 100 µL of the sample was spread onto an MHA plate with a sterile spreader, then incubated at 37°C overnight. The bacterial culture count was performed the next day using the colony-counting method.

For data analysis in this study, GraphPad Prism version 10.2.3 (GraphPad Software, La Jolla, CA, USA) was used. An unpaired t-test, a two-tailed Student’s t-test, and the Mann-Whitney U-test were used for the determination of the significance of the difference in the viable bacterial counts between the different groups. Data were considered statistically significant at p < 0.05.

## Results

**Figure 3:**
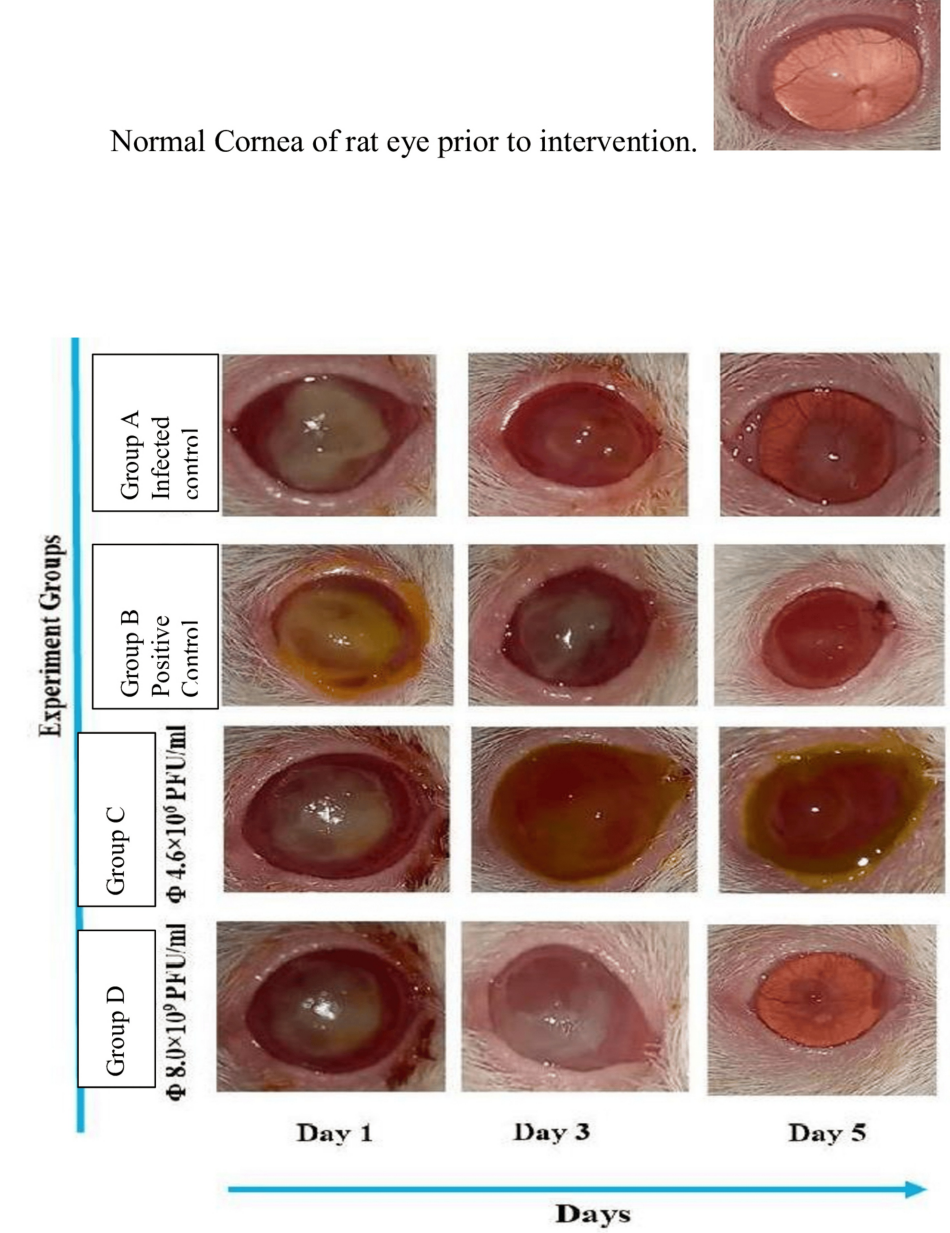
Effects of two different concentrations of bacteriophage dose administration as topical phage against Pseudomonas aeruginosa on the clinical signs of keratitis in the infected mice. All experimental groups and controls were noted and analysed day-wise, with their respective treatment doses. PFU: plaque-forming units

In our study, the efficacy of bacteriophages in destroying *Pseudomonas aeruginosa* in vivo was evaluated using an ulcer model in mice. All clinical findings in the mice across all groups were analysed by an ophthalmologist. The day-wise analysis is shown in Figure 3.

Following *Pseudomonas aeruginosa* 11434 infection of the cornea, a ring was visible on the first day, and by the second day, the whole cornea was covered with oedema. On day 5 post infection, we saw that the corneas of the mice in group A (infected control group), which were given PBS without phage as a blank treatment, were perforated. On the other hand, the corneas of the mice of group B (positive control), which were given four doses of antibiotics (ciprofloxacin 0.3%), displayed full corneal oedema on day 1 of post-corneal infection, which gradually disappeared by day 5. The test groups (group C and group D), which were given two concentrations, viz., low and high: phage 1 (4.6×10⁶ PFU/ml) and phage 2 (8.0×10⁹ PFU/ml), respectively, when compared to group B that received antibiotic treatment, phage 1 (group C mice) demonstrated extremely low clearance on day 5 after infection. On the other hand, phage 2 (group D mice) displayed significant clearing of corneal oedema, nearly identical to that observed with antibiotics. Based on the clinical score, therapy with phage 2 led to a considerably better illness outcome on days 1, 3, and 5 post infection. It is also considered a potentially viable treatment option for bacteria resistant to many drugs.

Estimation of the number of live bacteria in the corneal infection on MHA plates

To verify the emergence of phage resistance and the phages' capacity to lyse *Pseudomonas aeruginosa*, we conducted an experiment in which phage and bacteria were co-cultured. To count bacterial numbers daily, we first collected bacterial samples from all affected groups and cultured them on MHA plates. Table 1 shows the number of bacteria present in each millilitre of solution day-wise. 

**Table 1:** Study of bacterial load during the experimental duration in colony-forming units (CFUs)/mL

	Group A	Group B	Group C Bacteriophage 1	Group D Bacteriophage 2
Day	Infected Control (CFU/ml)	Positive Control (CFU/ml)	(Conc.4.6×106 PFU/ml)(CFU/ml)	(Conc.8.0×109PFU/ml) (CFU/ml)
Day 1	7083.33	7166.66	7033.33	7116.66
Day 2	9303.33	9516.66	9400.00	9153.33
Day 3	8106.66	7556.66	7733.33	7263.33
Day 4	7103.33	5113.33	5356.66	5043.33
Day 5	5576.66	3003.33	3123.33	2796.66
Mean	7434.66	6471.33	6529.33	6274.66

According to the ANOVA approach, the significant p-value in this table, which verified the experiment's significance, was p<0.05 (Table 1). The relationship between bacterial load (CFU/ml) and day, as well as the mean bacterial load (CFU/ml) across treatment groups, was depicted in these two graphs. From the first to the fifth day after infection, the groups treated with antibiotics and phage 2 had nearly identical levels of effectiveness. The phage 1-treated group was extremely sluggish but effective. Statistical analysis was performed, and the data of ANOVA showed that the F value was 11.55, and the p-value was <0.0001, with the R-squared (R^2^) value found to be 0.6979.

Histopathophysiology

**Figure 4:**
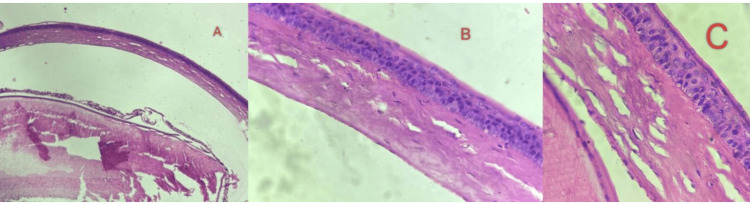
Histopathology of infected corneas in various experimental groups with different concentrations of bacteriophages: (A) control, (B) disease control, and (C) phage treatment.

Histopathology analysis suggested that on day 5 post infection, corneas from vehicle, positive control, and phage-treated mice were examined and analysed. Large neutrophilic abscesses, central corneal stroma weakening and oedema, and denuded epithelium were all visible in the corneas of mice given the vehicle. Picrosirius red staining of the mock-treated animals showed that the cornea's stromal structure had been disrupted and that the central region of the cornea had very few stromal collagen fibrils, as shown in Figure 4A.

Mice treated with ciprofloxacin 0.3% w/v as a positive control had normal corneal structure. On the other hand, mice treated with phage exhibited nearly normal corneal structure in both the treated groups (Figures 4B, 4C). *Pseudomonas aeruginosa* was found within the abscess in the mock-treated mice's corneas, according to the examination of mAb-labelled histological sections; in the positive control and phage-treated corneas, bacteria were hardly noticeable. At day 5 post infection, the bacterial load in the corneas of mice treated with the vehicle was considerably higher than that in the animals treated with bacteriophages and the positive controls. In phage-treated groups of mice, normal corneal structure was maintained, and no inflammatory cells were present.

## Discussion

We demonstrated the in vivo mouse model in this study. Before starting the mouse model, we standardised it by specifying anaesthesia duration, bacterial dose, eye scratching, therapeutic doses, etc. After that, we performed our main study, which included four groups: control, positive control, and two different concentration test groups. Finally, we assessed the bacteriophage's efficacy compared to antibiotics [9].

In an in vivo model of *Pseudomonas aeruginosa*-induced ulceration, the current study demonstrated that the four doses of phage 2 effectively removed bacteria from the diseased cornea, comparable to antibiotics. As a consequence, the disease's prognosis was improved, the cornea's oedema, opacities, and structure were avoided, and neutrophil activity was suppressed. This finding suggests that bacteriophages could be a unique treatment for antibiotic-resistant bacterial ulcers [10].

The positive control groups demonstrated severe corneal ulceration, confirming the validity of the model. Antibiotic treatment partially restored corneal tissue integrity, indicating its therapeutic efficacy. Bacteriophage treatment at both concentrations showed dose-dependent improvements in histopathological features, with the higher concentration approaching or surpassing the effects of antibiotic treatment. These results suggest the potential of bacteriophage therapy as a promising intervention for *Pseudomonas aeruginosa*-induced corneal ulcers, warranting further investigation to establish its clinical utility.

Histopathological analysis of corneal tissues from different treatment groups following a five-day treatment period in a mouse model of *Pseudomonas aeruginosa*-induced corneal ulcer revealed distinct patterns of tissue response.

Placebo and disease control groups showed severe corneal damage, inflammatory cell infiltration, vascular changes, and tissue necrosis consistent with an untreated corneal ulcer. In the antibiotic group, there was partial restoration of tissue integrity, reduced inflammation, and tissue necrosis, indicating therapeutic efficacy. Bacteriophage test groups showed dose-dependent improvement in histopathological features, with the higher phage concentrations showing significant restoration of tissue integrity and reduction in inflammation [11].

The study's short-term nature (five days) may not fully capture the long-term effects and durability of bacteriophage therapy. Mechanistic studies, including molecular pathways involved in bacteriophage-mediated tissue healing, are needed for a comprehensive understanding. Clinical trials involving human subjects with corneal ulcers are essential to validate these findings and establish guidelines for the use of bacteriophages in ophthalmic infections.

## Conclusions

Bacteriophage therapy shows promising results in mitigating *Pseudomonas aeruginosa*-induced corneal ulcers by clearing bacteria, exerting anti-inflammatory effects, and promoting tissue repair. This study on mice shows promising results in treating *Pseudomonas aeruginosa*-induced corneal ulcers, leading a path for its use as an independent drug topically or as an adjuvant therapy with antibiotics. 

Further research and clinical trials are necessary to translate these findings into effective therapeutic strategies for improving patient outcomes in ophthalmic infections. Studies based on the long-term effects of bacteriophage therapy, its side effects (short-term and long-term), and any harmful effects on long-term use still need to be done and validated.

In the future, if bacteriophage therapy succeeds in human trials and guidelines are established for its use in ophthalmic infections, then it will be a boon in this medical world where antibiotic resistance is rapidly increasing. This therapy, in the future, can serve the entire humanity.
